# Prognostic nutritional index and inflammatory indices as predictors of perioperative infection in patients with Cushing syndrome: a retrospective single-center study

**DOI:** 10.3389/fendo.2026.1798032

**Published:** 2026-04-24

**Authors:** Onour Chasan, Mehves Beliz Besisik Terzioglu, Ummu Mutlu, Duygu Dolen Burak, Hulya Hacisahinogullari, Nurdan Gul, Ozlem Soyluk Selcukbiricik, Ayse Kubat Uzum, Gulsah Yenidunya Yalin

**Affiliations:** 1 Division of Endocrinology and Metabolism, Department of Internal Medicine, Istanbul Faculty of Medicine, Istanbul University, Istanbul, Türkiye; 2 Department of Internal Medicine, Istanbul Faculty of Medicine, Istanbul University, Istanbul, Türkiye; 3 Department of Neurosurgery, Istanbul Faculty of Medicine, Istanbul University, Istanbul, Türkiye

**Keywords:** cortisol, Cushing syndrome (CS), dexamethasone suppression test (DST), infections, inflammatory indices, prognostic nutrition index (PNI)

## Abstract

**Objective:**

Endogenous Cushing syndrome (CS) is associated with increased risk of morbidity and mortality, and infections are among its major complications. The aim of this study was to evaluate the predictive value of cortisol-related indices, blood cell-derived inflammatory indices, and the prognostic nutritional index (PNI) for perioperative infection and to identify risk factors.

**Methods:**

A total of 113 patients with confirmed CS were included: 71 pituitary CS, 37 adrenal CS, and 5 ectopic CS. ROC analysis was performed to evaluate predictors of infection. Multivariable models were constructed, and the best-performing model was selected as the final model.

**Results:**

Thirty-five CS patients (31%) developed infection. ROC analysis demonstrated that 1-mg DST (AUC = 0.852) and PNI (AUC = 0.845) were the strongest predictors of infection. In the final model, 1-mg DST ≥ 17.2 µg/dL (OR: 9.741; 95% CI: 2.801-33.883; P < 0.001), PNI ≤ 51.4 (OR: 9.569; 95% CI: 2.683-34.122; P < 0.001), the presence of diabetes mellitus (OR: 3.963; 95% CI: 1.128-13.925; P = 0.032), and presence of bone fracture(s) or a T-score ≤ -3 (OR: 3.574; 95% CI: 1.003-12.730; P = 0.049) were identified as independent risk factors for infection.

**Conclusion:**

Perioperative infection was associated with elevated cortisol and lower PNI levels in patients with CS. Assessment of 1-mg DST and PNI, in conjunction with diabetes mellitus status and bone health, may enhance the identification of high-risk patients and guide targeted preventive strategies.

## 1 Introduction

Endogenous Cushing syndrome (CS) is associated with substantial morbidity and mortality, largely driven by cardiovascular, infectious, and thromboembolic complications (1–4). In addition, diabetes mellitus (DM), hypertension (HT), osteoporosis, and fragility fractures are common cortisol-related comorbidities (5, 6).

The risk of infection is markedly increased in patients with CS compared with the general population, and infections represent an important cause of death in this population (1–3). Excess cortisol impairs both innate and adaptive immune responses, resulting in lymphopenia, altered leukocyte function, and increased susceptibility to bacterial, viral, and fungal infections (7, 8). Importantly, this increased risk of infectious morbidity and mortality may not normalize immediately after curative surgery, because recovery from hypercortisolism-related immune and metabolic abnormalities may be delayed or incomplete (1).

Endogenous CS can be classified as ACTH-dependent (Cushing disease due to pituitary hypersecretion of ACTH or ectopic CS due to ectopic secretion of ACTH or CRH by non-pituitary tumors) or ACTH-independent (excessive cortisol secretion by the adrenal glands) (5, 6). Among the etiological subtypes, ectopic Cushing syndrome is typically characterized by the highest cortisol burden and a particularly high frequency of severe complications, including infection (1). It is also associated with high mortality and poor prognosis (3). Although cortisol-related severity is clinically relevant, data integrating hormonal burden with blood cell-derived inflammatory indices and nutritional-immune markers for the prediction of perioperative infection in CS remain limited. Therefore, we aimed to evaluate the predictive value of cortisol-related indices, blood cell-derived inflammatory indices, and the prognostic nutritional index (PNI) for perioperative infection in patients with CS, and to identify independent clinical risk factors associated with infection development.

## 2 Methods

### 2.1 Patient selection and study design

**Figure 1:**
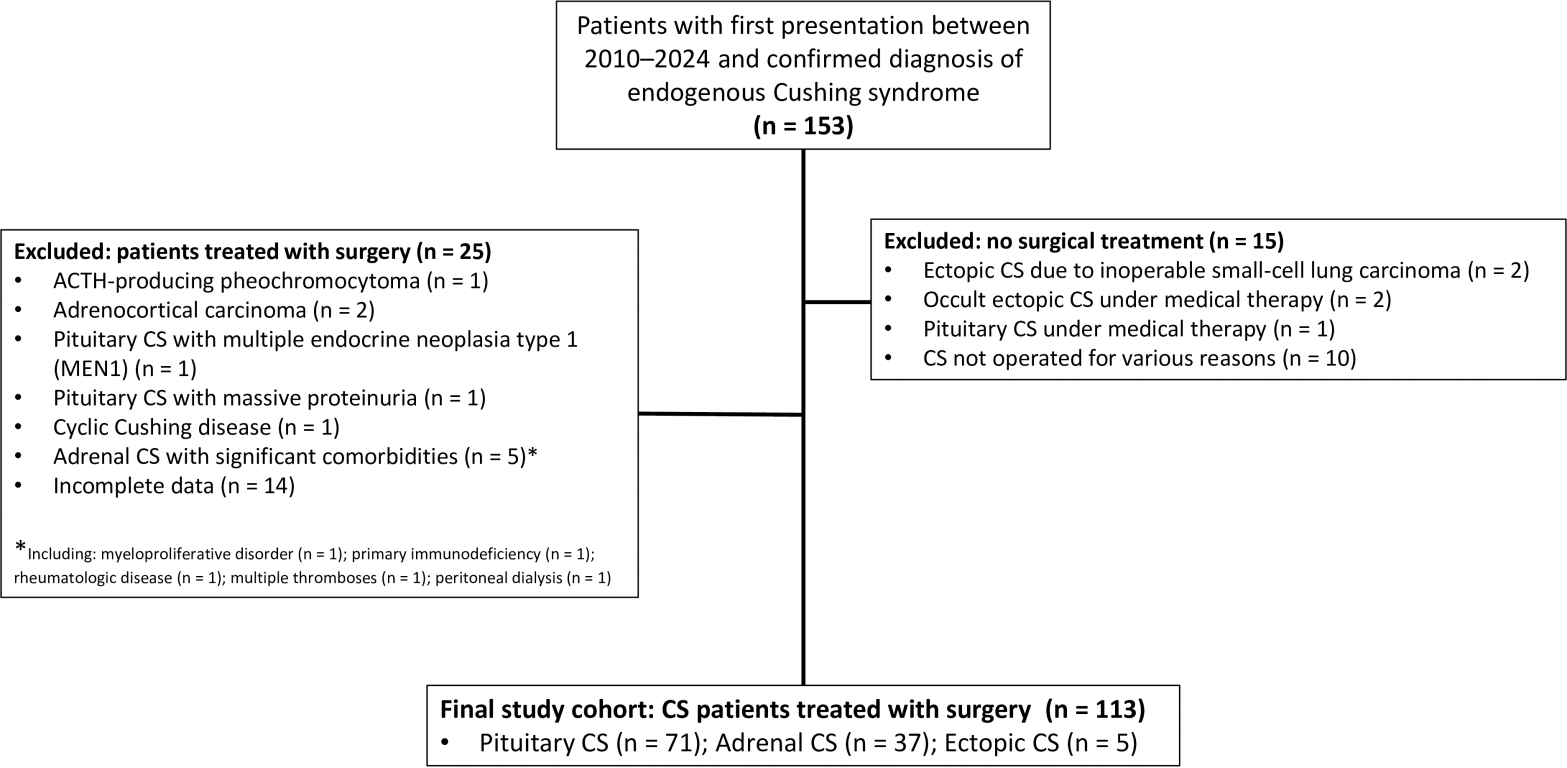
Flowchart of patient selection. CS, Cushing syndrome.

In this retrospective single-center study, the clinical and laboratory data of patients with endogenous CS followed at the Endocrinology and Metabolism Clinic between 2010 and 2024 were analyzed. All included patients had clinically and biochemically confirmed endogenous CS and underwent surgical treatment after standard etiological evaluation. A total of 153 patients were identified. Fifteen patients who did not undergo surgery were excluded, fourteen patients were excluded because of incomplete perioperative data, and eleven additional patients were excluded because they had conditions that could substantially affect hematological, inflammatory, or nutritional laboratory parameters, as shown in **Figure 1**. These conditions included hematologic disorders, active malignancy, and other clinically relevant conditions known to influence blood counts, albumin levels, or inflammatory indices. A total of 113 patients with CS were included in this study, comprising 71 with pituitary CS (Pit-CS), 37 with adrenal CS (Adr-CS), and 5 with ectopic CS (Ect-CS) (**Figure 1**). Data were analyzed based on each patient’s first surgery.

Clinical features and laboratory parameters were extracted from archived medical records. At diagnosis, body mass index (BMI), bone mineral density (BMD), and hematological and biochemical parameters were recorded. In addition, the presence of DM, HT, dyslipidemia, and hypokalemia was noted. Complications, including acute myocardial infarction (AMI), thromboembolism (pulmonary embolism [PE] or deep vein thrombosis [DVT]), ischemic or hemorrhagic cerebrovascular events, bone fractures, and avascular necrosis, were also documented. Laboratory parameters were evaluated before the dexamethasone suppression test (DST). In patients with preoperative infection, laboratory values obtained either before infection onset or after complete clinical recovery were used. Patients were categorized according to etiology as Pit-CS, Adr-CS, or Ect-CS. Comorbidities, complications, inflammatory indices, PNI, and cortisol-related indices were compared across etiological subgroups. Subsequently, clinical and biochemical parameters were compared between patients with and without perioperative infections.

### 2.2 Cushing syndrome-related data

In individuals presenting with clinical signs and symptoms suggestive of CS, the diagnosis was established if at least two of the following tests were positive (9, 10): serum cortisol >1.8 µg/dL after the overnight 1-mg DST (1-mg DST) or the low-dose DST (LDDST; 2-day, 2 mg/day); 24-hour urinary free cortisol (UFC) exceeding 1.5–2 times the upper limit of normal (ULN); late-night plasma cortisol (LNPC) >7.5 µg/dL; or elevated late-night salivary cortisol (LNSC) >0.145 µg/dL. For repeated measurements, the peak value was used for analysis.

Cortisol-related indices were reported as follows: basal cortisol levels, cortisol levels after the 1-mg DST, LDDST, and LNPC were reported in µg/dL. UFC and LNSC levels were expressed as the fold increase relative to the ULN (UFC/ULN ratio and LNSC/ULN ratio, respectively).

The differential diagnosis between adrenocorticotropic hormone (ACTH)-dependent and ACTH-independent Cushing syndrome was based on fasting ACTH levels. To distinguish between Ect-CS and Pit-CS, pituitary magnetic resonance imaging (MRI) and serum cortisol measurement after a high-dose overnight DST (8-mg DST) were used. When necessary, inferior petrosal sinus sampling, thoracic computed tomography (CT), abdominal CT or nuclear imaging were performed (10).

### 2.3 Inflammatory indices and prognostic nutritional index

Blood cell-derived inflammatory indices were calculated with the following formulas (11): Neutrophil-to-Lymphocyte Ratio (NLR) = Neutrophil count (10^9^/L)/Lymphocyte count (10^9^/L), Systemic Immune-Inflammation Index (SII) = Platelet count (10^9^/L) × Neutrophil count (10^9^/L)/Lymphocyte count (10^9^/L), Pan-Immune-Inflammation Value (PIV) = Neutrophil count (10^9^/L) × Platelet count (10^9^/L) × Monocyte count (10^9^/L)/Lymphocyte count (10^9^/L). PNI was calculated as Albumin (g/L) + 5 × Lymphocyte count (10^9^/L) (12).

### 2.4 Comorbidities and complications

DM was defined as follows (13): fasting blood glucose level ≥126 mg/dL or glucose level ≥200 mg/dL 2 hours after a 75-g oral glucose tolerance test (OGTT), or HbA1c ≥6.5%; HT (14): systolic blood pressure (SBP) ≥140 mmHg, diastolic blood pressure (DBP) ≥90 mmHg; and dyslipidemia (15): low-density lipoprotein cholesterol ≥130 mg/dL, or triglycerides ≥150 mg/dL. DVT was confirmed by venous compression Doppler ultrasound, and PE was diagnosed using computed tomography pulmonary angiography (CTPA) or ventilation/perfusion (V/Q) scintigraphy (16). AMI was diagnosed based on cardiac enzyme measurements, electrocardiogram findings, and coronary angiography (17). Ischemic cerebrovascular events were identified by diffusion-weighted MRI and/or CT angiography, whereas hemorrhagic cerebrovascular events were diagnosed by cranial MRI (18). All these diagnoses had been established before the study period and were obtained from existing medical records. BMD was assessed after diagnosis using a dual-energy X-ray absorptiometry (DXA) device. The lowest T-score among the lumbar spine (L1-L4), femoral neck, and total femur measurements were recorded. Bone fractures (vertebral or femoral) were identified using radiography, MRI, or CT (19). Hypokalemia was defined as a serum potassium level <3.5 mEq/L (reference range: 3.5-5.5 mEq/L).

### 2.5 Infections

Patients were categorized into two groups: with infection [INF (+)] and without infection [INF (–)]. Patients were assigned to the INF (+) group if they developed a clinically significant infection confirmed by laboratory and/or radiological evidence, accompanied by findings suggestive of systemic involvement and requiring treatment with oral or parenteral antimicrobial therapy. Localized fungal and other non-systemic localized infections were excluded to reduce heterogeneity and to avoid inflating the outcome definition with minor superficial infections. Excluded infections included superficial fungal infections such as tinea pedis, tinea corporis, tinea unguium, and tinea cruris, as well as mucosal candidiasis, scabies, vaginitis, blepharitis, conjunctivitis, and similar superficial infections. Infections were categorized according to their anatomical site of origin, because causative microorganisms were unavailable in most cases. Infections occurring from the time of diagnosis until surgery were classified as preoperative infections. Infections occurring within 3 months after surgery were classified as postoperative infections. For analysis, patients with one or more perioperative infections were categorized as INF (+) irrespective of the frequency or recurrence of infections, defining infection as a single binary outcome.

### 2.6 Statistical analyses

Statistical analyses were performed using IBM SPSS Statistics for Windows v30.0.0 (IBM Corp., Armonk, NY). Kolmogorov-Smirnov test and Shapiro-Wilk test were used as normality tests for the quantitative parameters. Comparisons of continuous variables between two groups were evaluated using Student t-tests for normally distributed values or the Mann-Whitney U-test for non-normally distributed values. For comparisons of continuous values across the three groups, one-way ANOVA was used for parametric values, while the Kruskal-Wallis test was used for non-parametric values. Results were expressed as mean (standard deviation) when normally distributed or as median (interquartile range) when non-normally distributed.

The Pearson chi-squared test or Fisher’s exact test was used to compare categorical variables with a 2×2 distribution, while the Fisher-Freeman-Halton exact test was applied for contingency tables larger than 2×2. All categorical test results were reported as percentages.

Relationships between variables were examined using Spearman correlation analysis for non-normally distributed data. Receiver operating characteristic (ROC) curve analysis was performed to evaluate the diagnostic performance of the selected variables, with the area under the curve (AUC) calculated, optimal cut-off values determined using the Youden index, and corresponding sensitivity, specificity, positive predictive value (PPV), and negative predictive value (NPV) reported.

Binary logistic regression analyses were performed to identify associated factors with perioperative infection. Variables with P < 0.20 in the univariable analysis were entered into a multivariable logistic regression model using a stepwise selection procedure. Final models were evaluated based on their discriminative ability using ROC curve analysis, and the model with the best discriminative performance was selected as the final model. All statistical analyses were two-sided, and P-values < 0.05 were considered statistically significant.

## 3 Results

### 3.1 Comparison of pituitary, adrenal, and ectopic etiologies

One hundred and thirteen patients with CS were included in this study (18 males/95 females). Among the study population, the etiology of CS was Pit-CS in 71 patients (63%), Adr-CS in 37 patients (33%), and Ect-CS in 5 patients (4%). All Ect-CS were caused by ACTH/corticotropin releasing hormone (CRH)-secreting pulmonary neuroendocrine tumors. The baseline characteristics, cortisol-related indices, blood cell-derived inflammatory indices, PNI values, comorbidities, and complications of the patients are shown in **Table 1**. Males predominated in the Ect-CS (3/5, 60%; P = 0.039). Median age at diagnosis was 43 years in Pit-CS, 52 in Adr-CS, and 38 in Ect-CS (P = 0.059). The median duration from symptom onset to diagnosis was shorter in Ect-CS compared with in Pit-CS (9.5 [6-20.5] vs 42 [18-60] month, respectively; (P = 0.044) (**Table 1**).

**Table 1:** Clinical and laboratory characteristics of patients with endogenous Cushing syndrome: comparison of pituitary, adrenal, and ectopic etiologies.

	All patients	Pit-CS	Adr-CS	Ect-CS	P*-*value
Variables	(n=113)	(n=71)	(n=37)	(n=5)	
Baseline characteristics					
Sex, male, n (%)	18 (15.9%)	11 (15.5%)^c^	4 (10.8%)^c^	3 (60%)^a,b^	**0.039**
Age at diagnosis, year, median (IQR)	44 (33–55)*	43 (33–51)*	52 (34.5–60)*	38 (28–49)*	0.059
BMI, kg/m²,mean ± SD	33.36 ± 5.89	34.30 ± 6.01	31.80 ± 4.81	31.95 ± 8.75	0.287
From symptom onset to diagnosis, month, median (IQR)	36 (12–60)*	42 (18–60)*^c^	36 (12–66)*	9.5 (6–20.5)*^a^	**0.044**
From diagnosis to surgery, month, median (IQR)	5 (3–10)*	6 (3–11.5)*	4 (2–9.75)*	3.5 (2.25–4.75)*	0.146
Cortisol-related indices					
Basal cortisol, µg/dL, median (IQR)	20.66 (16.45-25.18)* (n = 106)	20.23 (16.63–26.99)*^c^ (n = 68)	19.53 (15.29–23.08)*^c^ (n = 33)	29.75 (22.42-46.28)^a,b^ (n = 5)	**0.032**
1-mg DST, µg/dL, mean ± SD	16.45 ± 10.66	16.58 ± 10.58^c^	13.55 ± 8.00 ^c^	33.91 ± 12.08^a,b^	**<0.001**
LDDST, µg/dL, median (IQR)	11.35 (6.95-19.83)* (n = 104)	12 (7.25-19.50)*^c^ (n = 63)	10.15 (5.83-18.3)*^c^ (n = 36)	25 (16.57 -34.7)*^a,b^ (n = 5)	**0.033**
LNPC, µg/dL, mean ± SD	18.2 ± 11.99 (n = 28)	15.13 ± 4.11 ^c^ (n = 19)	15.19 ± 5.55 ^c^ (n = 6)	43.67 ± 24.99 a^,b^ (n = 3)	**<0.001**
LNSC, xULN, median (IQR)	3.37 (1.70–6.62)* (n = 40)	3.37 (1.73–5.56)*^c^ (n = 26)	2.15 (1.53–4.27)*^c^ (n = 11)	18 (7.35-41.10)*^a,b^ (n = 3)	**0.029**
UFC, xULN, median (IQR)	2.18 (1.47–5.37)* (n = 38)	2.16 (1.49–3.91)*^c^ (n = 24)	1.85 (1.27–3.83)*^c^ (n = 10)	9.65 (7.08-13.5)*^a,b^ (n = 4)	**0.012**
PNI (mean ± SD)	54.37 ± 5.97	54.40 ± 5.96	55.00 ± 6.00^c^	49.27 ± 3.85^b^	0.045
Blood-cell-derived inflammatory indices					
NLR,(mean ± SD)	3.45 ± 1.56	3.56 ± 1.53^c^	2.96 ± 1.36 ^c^	5.33 ± 1.91^a,b^	**0.003**
SII, (mean ± SD)	981.40 ± 519.94	1020 ± 509	847 ± 471 ^c^	1432 ± 765 ^b^	**0.035**
PIV, median (IQR)	480 (269.5–1005)*	650 (339–1099)*	460 (275–814)*	972 (508–1223)*	0.075
Comorbidities, n (%)					
Diabetes mellitus	55 (48.7%)	35 (49.3%)	17 (45.9%)	3 (60%)	0.849
Hypertension	81 (71.7%)	50 (70.4%)	27 (73.0%)	4 (80%)	0.935
Dyslipidemia	75 (66.4%)	48 (67.6%)	23 (62.2%)	4 (80%)	0.731
Bone complications, n (%)					
Bone fracture(s)	16 (14.2%)	11 (15.5%)	3 (8.1%)	2 (40%)	0.141
Bone fracture(s) or a T-score ≤ –3	34 (30.1%)	22 (31.0%)	10 (27.0%)	2 (40%)	0.355
T-score > −3 and ≤ -1 (no fracture)	37 (32.7%)	26 (36.6%)	9 (24.3%)	2 (40%)	0.438
Avascular necrosis	5 (4.4%)	3 (4.2%)	2 (5.4%)	0 (0%)	0.764
Cardiac and vascular complications, n (%)					
Presence of ≥ 1 cardiac or vascular complications	18 (15.9%)	9 (12.7%)	8 (21.6%)	1 (20%)	0.363
Acute myocardial infarction	15 (13.3%)	8 (11.3%)	6 (16.2%)	1 (20%)	0.528
Deep vein thrombosis	3 (2.7%)	1 (1.4%)	1 (2.7%)	1 (20%)	0.159
Pulmonary embolism	1 (0.9%)	1 (1.4%)	0 (0%)	0 (0%)	0.627
Ischemic cerebrovascular event	4 (3.5%)	2 (2.8%)	2 (5.4%)	0 (0%)	0.673
Hemorrhagic cerebrovascular event	2 (1.8%)	1 (1.4%)	1 (2.7%)	0 (0%)	0.821
Hypokalemia, n (%)	9 (8.0%)	5 (7.0%)^c^	1 (2.7%)^c^	3 (60.0%)^a,b^	**0.004**
Infection, n (%)					
Presence of ≥ 1 perioperative infection	35 (31%)	24 (33.8%)	7 (18.9%)^c^	4 (80%)^b^	**0.013**
Preoperative infection	20 (17.7%)	13 (18.3%)	4 (10.8%)^c^	3 (60%)^b^	**0.038**
Postoperative infection	27 (23.9%)	19 (26.8%)^c^	4 (10.8%)^c^	4 (80%)^a,b^	**0.003**

Pit-CS, pituitary-dependent Cushing syndrome; Adr-CS, Adrenal-dependent Cushing syndrome; Ect-CS, Cushing syndrome from ectopic source; BMI, body mass index; 1-mg DST, overnight 1-mg dexamethasone suppression test; LDDST, longer low-dose dexamethasone suppression test; LNPC, late-night plasma cortisol; LNSC, late-night salivary cortisol; UFC, 24-h urinary free cortisol; ULN, upper limit of normal; NLR, neutrophil-to-lymphocyte ratio; SII, systemic immune-inflammation index; PIV, Pan-immune-inflammation value; PNI, prognostic nutritional index (PNI).

Shown are mean ± SD for normally distributed variables, median (interquartile range, IQR) for non-normally distributed variables*, and n (%) for absolute numbers. For comparisons of continuous variables, one-way ANOVA was used for normal distributed values; while the Kruskal–Wallis test was used for non-normally distributed variables; followed by *post-hoc* analyses. Categorical variables were analyzed using the Fisher–Freeman–Halton exact test. Values in bold indicate statistical significance, with P-values less than 0.05.

*non-normally distributed data.

^a^ P ≤ 0.05 vs Pit-CS, ^b^P ≤0.05 vs Adr-CS, ^c^P ≤.0.05 vs Ect-CS.

Basal cortisol level was higher in Ect-CS than in Pit-CS and Adr-CS (29.75 [22.42-46.28] vs 20.23 [16.63-26.99], and 19.53 [15.29-23.08] µg/dL, respectively; P = 0.032). The 1-mg DST was higher in the Ect-CS than in Pit-CS and Adr-CS (33.91 ± 12.08 vs 16.58 ± 10.58, and 13.55 ± 8.00 µg/dL, respectively; P < 0.001). Similarly, LDDST was higher in Ect-CS than in Pit-CS and Adr-CS (25 [16.57-34.7], vs 12 [7.25-19.50], and 10.15 [5.83-18.30] µg/dL, respectively; P < 0.001) (**Table 1**).

PNI was lower in Ect-CS than in Adr-CS (49.27 ± 3.85 vs 55.00 ± 6.00; P = 0.034) (**Table 1**). Regarding the blood cell-derived inflammatory indices, NLR was higher in Ect-CS compared with that in Pit-CS and Adr-CS (5.33 ± 1.91 vs 3.56± 1.53, and 2.96 ± 1.36, respectively; P = 0.003). SII was higher in Ect-CS than in Adr-CS (1432 ± 765 vs 847 ± 471; P = 0.035); other pairwise comparisons were non-significant (P > 0.05). PIV were similar among the groups (P > 0.05) (**Table 1**).

The frequency of DM, HT, and dyslipidemia did not differ among the groups (P > 0.05). In addition, no differences were observed in the frequency of cardiac and vascular complications (P > 0.05). Hypokalemia was more frequent in Ect-CS (3/5, 60%) than in Pit-CS (5/71, 7.0%) and in Adr-CS (1/37, 2.7%) (P = 0.004) (**Table 1**). Postoperative infection was more frequent in Ect-CS (4/5, 80%) than in Pit-CS (19/71, 26.8%) and in Adr-CS (4/37, 10.8%) (P = 0.003) (**Table 1**).

### 3.2 Comparison by perioperative infection status

Among the study population, perioperative infections occurred in 35 patients [INF (+) group, 31%]. The INF (+) comprised 69% Pit-CS (n=24), 20% Adr-CS (n=7), and 11% Ect-CS (n=4), whereas 78 patients with no perioperative infections [INF (-) group, 69%] comprised 60% Pit-CS (n=47), 39% Adr-CS (n=30), and 1% Ect-CS (n=1).

The mean age at diagnosis was similar between INF (+) and INF (-) (40.17 ± 13.62 vs. 44.47 ± 13.00, P = 0.101). BMI was also similar between INF (+) and INF (-) (33.38 [31.19-39.63] vs. 34.18 [28.55-37.59], P = 0.324) (**Table 2**).

**Table 2:** Comparison of clinical and laboratory parameters by perioperative infection status in patients with endogenous Cushing syndrome.

Variables	INF (–) (n=78)	INF (+) (n=35)	P*-*value
Baseline characteristics			
Sex, Male, n (%)	11 (14.1%)	7 (20.0%)	0.428
Age at diagnosis, year, mean ± SD	44.47 ± 13	40.17 ± 13.62	0.101
BMI, kg/m², median (IQR)	34.18 (28.55–37.59)*	33.38 (31.19–39.63)*	0.324
From symptom onset to diagnosis, month, median (IQR)	36 (12–54)*	33 (18–69)*	0.658
From diagnosis to surgery, month, median (IQR)	6 (3–15.5)*	4 (2–10)*	0.213
Cortisol-related indices			
Basal cortisol (08:00), µg/dL, median (IQR)	17.8 (13.26-22.69)* (n = 73)	21.46 (17.4-34)* (n = 33)	**<0.001**
1-mg DST, µg/dL, mean ± SD	12.6 (8.33–19.83)*	19.12 (17.43–21.6)*	**<0.001**
LDDST, µg/dL, median (IQR)	9.8 (5.41-14)* (n = 71)	15.3 (11.5-25.6)* (n = 33)	**<0.001**
LNPC, µg/dL,median (IQR)	15 (12.05-15.27)* (n = 17)	19.90 (15.90-24)* (n = 11)	**0.01**
LNSC, xULN, median (IQR)	2.01 (1.51-3.9)* (n = 26)	6.35 (4.03-7.53)* (n = 14)	**<0.001**
UFC, xULN, mean ± SD	2.03 ± 1.33 (n = 24)	6.57 ± 4.12 (n = 14)	**<0.001**
PNI, median (IQR)	55.75 (52.05–60.75)*	49.28 (45.34–53.83)*	<0.001
Blood cell-derived inflammatory indices			
NLR, median (IQR)	2.47 (1.66–4.82)*	4.27 (3.69–5.18)*	**<0.001**
SII, median (IQR)	601 (441–1423)*	1471 (1218–1741)*	**<0.001**
PIV, median (IQR)	427 (220–1205)*	1266 (760–1435)*	**<0.001**
Comorbidities, n(%)			
Diabetes mellitus	31 (39.7%)	24 (68.6%)	**0.005**
Hypertension	52 (66.7%)	29 (82.9%)	0.077
Dyslipidemia	51 (65.4%)	24 (68.6%)	0.74
Bone complications, n (%)			
Bone fracture(s)	5 (%6.4)	11 (%31.4)	**<0.001**
Bone fracture(s) or T-score ≤ –3	16 (20.5%)	18 (51.4%)	**<0.001**
T-score > −3 and ≤ −1 (no fracture)	24 (%30.8)	13 (%37.1)	0.504
Avascular necrosis	5 (%6.4)	0 (%0)	0.322
Cardiac and vascular complications, n (%)			
Presence of ≥ 1 cardiac or vascular complications	12 (%15.4)	6 (%17.1)	0.813
Acute myocardial infarction	9 (11.5%)	6 (17.1%)	0.417
Deep vein thrombosis	1 (1.3%)	2 (5.7%)	0.226
Pulmonary embolism	0 (0.0%)	1 (2.9%)	0.310
Ischemic cerebrovascular event	4 (5.1%)	0 (0.0%)	0.309
Hemorrhagic cerebrovascular event	1 (1.3%)	1 (2.9%)	0.525
Operated under medical cortisol-lowering treatment	4 (5.1%)	4 (11.4%)	0.251
Hypokalemia, n (%)	1 (1.3%)	8 (22.9%)	**<0.001**

INF (–), no perioperative infection; INF (+), presence of perioperative infection; BMI, body mass index; 1-mg DST, overnight 1-mg dexamethasone suppression test; LDDST, longer low-dose dexamethasone suppression test; LNPC, late-night plasma cortisol; LNSC, late-night salivary cortisol; UFC, 24-h urinary free cortisol; ULN, upper limit of normal; NLR, neutrophil-to-lymphocyte ratio; SII, systemic immune-inflammation index; PIV, pan-immune-inflammation value; PNI, prognostic nutritional index.

Shown are mean ± SD for normally distributed variables, median (interquartile range, IQR) for non-normally distributed variables*, and n (%) for categorical variables. For comparisons of continuous variables, Student’s t-test was used for normally distributed data, while the Mann–Whitney U test was used for non-normally distributed data. Categorical variables were analyzed using the chi-square test or Fisher’s exact test. Values in bold indicate statistical significance, with P-values less than 0.05.

*non-normally distributed data.

The 1-mg DST was significantly higher in INF (+) than in INF (-) (19.12 [17.43-21.60] vs. 12.6 [8.3-19.8], P < 0.001). PNI was significantly lower in INF (+) than in INF (-) (49.28 [45.34-53.83] vs. 55.75 [52.05-60.75], P < 0.001) (**Table 2**).

Additionally, blood cell-derived inflammatory indices were evaluated, NLR, SII, and PIV values were significantly higher in INF (+) than in INF (-), median (IQR) values for NLR, SII, and PIV were 4.27 (3.69-5.18) vs. 2.47 (1.66-4.82), 1471 (1218-1741) vs. 601 (441-1423), and 1266 (760-1435) vs. 427 (220-1205), respectively (all P < 0.001) (**Table 2**).

DM was more frequent in INF (+) (24/35, 68.6%) than in INF (-) (31/78, 39.7%) (P = 0.005). HT and dyslipidemia did not differ among the groups (P > 0.05). No differences occurred in cardiac and vascular complications (P > 0.05) (**Table 2**).

The frequency of bone fracture(s) or a T-score ≤ -3 was significantly higher in INF (+) (18/35, 51.4%) compared with that in INF (-) (16/78, 20.5%) (P < 0.001). Hypokalemia was more frequent in INF (+) (8/35, 22.9%) than in INF (-) (1/78, 1.3%) (P < 0.001) (**Table 2**).

### 3.3 Infection characteristics and clinical course

Among the 35 patients in the INF (+) group, 8 patients developed infection only in the preoperative period, 15 patients only in the postoperative period, and 12 patients in both periods. Postoperative infections occurred at a median of 7 days after surgery. The most common infections were urinary tract infections, followed by pneumonia. Three patients developed clinically significant fungal infections: two in the preoperative period, both in Pit-CS patients (Candida esophagitis and a sinonasal fungal ball), and one in the postoperative period in a patient with Ect-CS (Candida septicemia). In addition, one patient with Pit-CS had a preoperative thyroid Actinomyces ball (**Table 3**). A total of seven intensive care unit admission infection events occurred in six patients (2 Ect-CS, 4 Pit-CS, and 1 Adr-CS).

**Table 3:** Distribution and types of preoperative and postoperative infections in patients with endogenous Cushing syndrome.

Infection origin	Total (n)	Preoperative infection event (n)	Postoperative infection event (n)
Urinary tract infection	19	7	12
Pneumonia	13	5	8
Upper respiratory infection	6	2	4
Acute gastroenteritis	4	1	3
Soft tissue infection Total (n)	**3**	**3**	**0**
Cellulitis of the lower limb	2	2	0
Inguinal soft-tissue infection	1	1	0
Abdominal wall abscess Total (n)	**3**	**1**	**2**
Incisional abdominal wall abscess	2	1	1
Incisional inguinal abscess	1	0	1
Dental abscess	3	1	2
Meningitis	2	0	2
Spondylodiscitis	1	1	0
Empyema	1	0	1
Epididymitis	1	0	1
Unknown origin systemic infection	1	0	1
Actinomyces ball in thyroid	1	1	0
Fungal infections Total (n)	**3**	**2**	**1**
Sinonasal fungal ball	1	1	0
Candida esophagitis	1	1	0
Candida septicemia	1	0	1

### 3.4 Correlation analyses

PNI demonstrated significant negative correlations with all cortisol-related indices, particularly for 1-mg DST (ρ = -0.605, P < 0.001) and UFC (xULN) (ρ = -0.605, P < 0.001). Among the blood cell-derived indices, NLR demonstrated significant positive correlations with several cortisol-related indices, particularly UFC (xULN) (ρ = 0.735, P < 0.001), LDDST (ρ = 0.621, P < 0.001), and 1-mg DST (ρ = 0.589, P < 0.001). SII demonstrated positive correlations with several cortisol-related indices, most notably UFC (xULN) (ρ = 0.503, P = 0.001), LDDST (ρ = 0.484, P < 0.001), and 1-mg DST (ρ = 0.471, P < 0.001). PIV demonstrated positive correlations with UFC (xULN) (ρ = 0.453, P = 0.004), LDDST (ρ = 0.440, P < 0.001, and 1-mg DST (ρ = 0.409, P < 0.001) (**Table 4**).

**Table 4:** Correlation analysis of cortisol-related indices with blood cell-derived indices and PNI.

Variables	PNI		NLR		SII		PIV	
	ρ	P*-*value	ρ	P*-*value	ρ	P*-*value	ρ	P*-*value
Basal cortisol (n = 106)	-0.452	**<0.001**	0.46	**<.0.001**	0.264	**0.006**	0.202	**0.038**
1-mg DST	-0.605	**<0.001**	0.589	**<0.001**	0.471	**<0.001**	0.409	**<0.001**
LDDST (n = 104)	-0.582	**<0.001**	0.621	**<0.001**	0.484	**<0.001**	0.44	**<0.001**
LNPC (n = 28)	-0.508	**0.006**	0.399	0.035	0.146	0.46	0.056	0.779
LNSC (xULN) (n = 40)	-0.471	**0.002**	0.413	**0.008**	0.32	0.044	0.18	0.268
UFC (xULN) (n = 38)	-0.605	**<0.001**	0.735	**<0.001**	0.503	**0.001**	0.453	**0.004**

1-mg DST, overnight 1-mg dexamethasone suppression test; LDDST, longer low-dose dexamethasone suppression test; LNPC, late-night plasma cortisol; LNSC, late-night salivary cortisol; UFC, 24-h urinary free cortisol; ULN, upper limit of normal; NLR, neutrophil-to-lymphocyte ratio; SII, systemic immune-inflammation index; PIV, pan-immune-inflammation value; PNI, prognostic nutritional index.

Values in bold indicate statistical significance, with P-values less than 0.05.

### 3.5 Predictive value of 1-mg DST and inflammatory–nutritional indices for infection risk

The 1-mg DST and PNI had the strongest discriminative performance for identifying patients who developed infection (AUC = 0.852; cut-off ≥17.2 µg/dL; sensitivity = 0.86; specificity = 0.80; PPV = 0.66; NPV = 0.90; P < 0.001 and AUC = 0.845; cut-off ≤ 51.4; sensitivity = 0.69; specificity = 0.88; PPV = 0.73; NPV = 0.86; P ≤ 0.001, respectively). NLR, SII, and PIV were also found to have acceptable discriminative performance for the prediction of infection development (**Table 5**).

**Table 5:** Receiver operating characteristic analysis of perioperative infection predictors in patients with endogenous Cushing syndrome.

Variables	AUC	Cut Off	Sens.	Spec.	PPV	NPV	P*-*value
Basal cortisol (n = 106)	0.714	19.185	0.788	0.548	0.44	0.85	**<0.001**
1-mg DST	0.852	17.2	0.86	0.8	0.66	0.9	**<0.001**
LDDST (n = 104)	0.809	11.15	0.909	0.648	0.54	0.938	**<0.001**
LNPC (n = 28)	0.791	15.6	0.818	0.824	0.75	0.875	**0.003**
LNSC (xULN) (n = 40)	0.812	2.475	0.929	0.654	0.59	0.944	**<0.001**
UFC (xULN) (n = 38)	0.839	3.65	0.714	0.917	0.83	0.846	**<0.001**
PNI^†^	0.845	51.4	0.69	0.88	0.73	0.86	**<0.001**
NLR	0.81	3.43	0.87	0.67	0.54	0.91	**<0.001**
SII	0.791	1141	0.66	0.78	0.67	0.84	**<0.001**
PIV	0.747	745.5	0.67	0.72	0.51	0.82	**<0.001**

AUC, area under the curve; Sens., sensitivity; Spec., specificity; PPV, positive predictive value; NPV, negative predictive value; NLR, neutrophil-to-lymphocyte ratio; SII, systemic immune-inflammation index; PIV, pan-immune-inflammation value; PNI, prognostic nutritional index; 1-mg DST, overnight 1-mg dexamethasone suppression test; LDDST, longer low-dose dexamethasone suppression test; LNPC, late-night plasma cortisol; LNSC, late-night salivary cortisol; UFC, 24-h urinary free cortisol; ULN, upper limit of normal. Cut-off values were determined using the Youden index. For risk (positive) classification, high-risk was defined as values ≥ cut-off for basal cortisol, 1-mg DST, LDDST, LNPC, LNSC (×ULN), UFC (×ULN), NLR, SII and PIV.

^†^ For PNI, high-risk was defined as values ≤ cut-off (lower PNI indicates higher risk).

Values in bold indicate statistical significance, with P-values less than 0.05.

### 3.6 Univariable and multivariable regression analyses

In the univariable analysis, lower PNI was significantly associated with perioperative infection (OR per 1-unit increase = 0.707; equivalently, OR per 1-unit decrease = 1.41, P < 0.001), while higher inflammatory indices, including NLR (OR = 2.446), SII (OR = 1.002), and PIV (OR = 1.002), were also significantly associated with perioperative infection (all P < 0.001). Elevated 1-mg DST (OR = 1.175; P < 0.001), DM (OR = 3.308; P = 0.006), and bone fracture(s) or a T-score ≤ -3 (OR = 4.602; P = 0.001) were further significantly associated with perioperative infection. Taken together, both cortisol-related indices and blood cell-derived inflammatory indices were significantly associated with perioperative infection in the univariable analysis. Based on the ROC-derived cut-off values, univariable analysis showed that PNI ≤ 51.4 and 1-mg DST ≥ 17.2 µg/dL were significantly associated with perioperative infection, with odds ratios (OR) of 16.727 (95% CI: 6.179-45.238; P < 0.001) and 17.722 (95% CI: 6.213-50.551; P < 0.001), respectively (**Table 6**).

**Table 6:** Univariable logistic regression analysis of clinical and laboratory parameters for perioperative infection in patients with endogenous Cushing syndrome.

Variables	OR (95% CI)	P*-*value
PNI ≤ 51.4	16.727 (6.179-45.238)	**<0.001**
1-mg DST ≥ 17.2	17.722 (6.213-50.551)	**<0.001**
Baseline characteristics		
Sex, male	1.523 (0.535–4.331)	0.430
Age at diagnosis	0.975 (0.946–1.006)	0.102
BMI	1.054 (0.956–1.163)	0.293
From symptom onset to diagnosis	1.005 (0.991–1.019)	0.493
From diagnosis to surgery	0.973 (0.936–1.010)	0.148
Cortisol-related indices		
Basal cortisol (n = 106)	1.113 (1.049-1.182)	**<0.001**
1-mg DST	1.175 (1.097–1.258)	**<0.001**
LDDST (n = 104)	1.145 (1.077-1.217)	**<0.001**
LNPC (n = 28)	1.220 (0.993-1.498)	0.058
LNSC (xULN) (n = 40)	1.541 (1.117-2.125)	**0.008**
UFC (xULN) (n = 38)	1.936 (1.244-3.011)	**0.003**
PNI	0.707 (0.612–0.816)	<0.001
Blood cell-derived inflammatory indices		
NLR	2.446 (1.681–3.560)	**<0.001**
SII	1.002 (1.001–1.004)	**<0.001**
PIV	1.002 (1.001–1.003)	**<0.001**
Comorbidities		
Diabetes mellitus	3.308 (1.420–7.705)	**0.006**
Hypertension	2.417 (0.892–6.551)	0.083
Dyslipidemia	1.155 (0.492–2.709)	0.74
Bone complications		
Bone fracture(s)	6.692 (2.112–21.204)	**0.001**
Bone fracture(s) or a T-score ≤ –3	4.103 (1.734–9.706)	**0.001**
T-score > −3 and ≤ −1 (no fracture)	1.330 (0.575-3.072)	0.505
Avascular necrosis	0 (0–—)	0.999
Cardiac and vascular complications		
Presence of ≥1 cardiac or vascular complications	1.138 (0.389-3.327)	0.813
Acute myocardial infarction	1.586 (0.517–4.863)	0.420
Deep vein thrombosis	4.667 (0.409–53.266)	0.215
Pulmonary embolism	3706089345 (0–—)	1
Ischemic cerebrovascular event	0 (0–—)	0.999
Hemorrhagic cerebrovascular event	2.265 (0.138–37.281)	0.567
Hypokalemia	22.815 (2.72-191)	**0.004**

CI, Confidence interval; OR, estimated odds ratio; 1-mg DST, overnight 1-mg dexamethasone suppression test; BMI, body mass index; LDDST, longer low-dose dexamethasone suppression test; LNPC, late-night plasma cortisol; LNSC, late-night salivary cortisol; UFC, 24-h urinary free cortisol; ULN, upper limit of normal; NLR, neutrophil-to-lymphocyte ratio; SII, systemic immune-inflammation index; PIV, pan-immune-inflammation value; PNI, prognostic nutritional index.

Values in bold indicate statistical significance, with P-values less than 0.05.

Variables with P < 0.20 in univariable logistic regression analyses were entered into the multivariable model using stepwise selection. In the final multivariable logistic regression model, PNI ≤ 51.4 (OR = 9.569, 95% CI: 2.683-34.122; P < 0.001), 1-mg DST ≥ 17.2 µg/dL (OR = 9.741, 95% CI: 2.801-33.883; P < 0.001), diabetes mellitus (OR = 3.963, 95% CI: 1.128-13.925; P = 0.032), and bone fracture(s) or a T-score ≤ -3 (OR = 3.574, 95% CI: 1.003-12.730; P = 0.049) were identified as independent risk factors for perioperative infection (**Table 7**). The model showed strong explanatory performance (Nagelkerke R² = 0.625), suggesting substantial predictive capacity, and excellent discrimination on ROC analysis (AUC = 0.921).

**Table 7:** Final multivariable logistic regression model identifying independent risk factors for perioperative infection in patients with endogenous Cushing syndrome.

Variables	Estimated regression coefficient	Estimated standard error	z	P*-* value	OR	95% Confidence interval of OR
PNI ≤ 51.4	2.259	0.649	3.48	**<0.001**	9.569	2.683-34.122
1-mg DST ≥ 17.2	2.276	0.636	3.58	**<0.001**	9.741	2.801-33.883
Diabetes mellitus	1.377	0.641	2.15	**0.032**	3.963	1.128-13.925
Bone fracture(s) or a T-score ≤ –3	1.274	0.648	1.97	**0.049**	3.574	1.003-12.730

1-mg DST, overnight 1-mg dexamethasone suppression test; PNI, prognostic nutritional index; OR, estimated odds ratio.

Values in bold indicate statistical significance, with P-values less than 0.05.

## 4 Discussion

In this retrospective single-center study, we evaluated perioperative infection risk in patients with endogenous CS using cortisol-related indices, blood cell-derived inflammatory markers, PNI, and selected clinical variables. Our main findings were that 1-mg DST and PNI showed the best discriminative performance for perioperative infection, and that 1-mg DST ≥17.2 µg/dL, PNI ≤51.4, DM, and severe bone involvement remained independently associated with infection risk in the final multivariable model. Patients with Ect-CS had the highest cortisol-related indices and the highest frequency of infection in our cohort. This is consistent with the recognized clinical severity of ectopic hypercortisolism, in which very high cortisol exposure is associated with a greater burden of acute complications, including infection (1). However, because the number of Ect-CS cases in our cohort was small, subgroup comparisons should be interpreted cautiously.

The observed association between PNI and infection risk is also supported by pathophysiological mechanisms. Glucocorticoid excess has been associated with lymphopenia through reduced lymphocyte proliferation, redistribution, and apoptosis, while simultaneously altering neutrophil trafficking and impairing immune defense (7, 8). In patients with CS, hypercortisolism has also been linked to alterations in serum protein levels, including decreased albumin levels (20). This decrease in albumin may be interpreted not as a direct mechanism contributing to infection development, but rather as a marker of impaired nutritional and metabolic status. Taken together, these mechanisms suggest that hypercortisolism may negatively affect the components of PNI, thereby providing a biological basis for the observed association between lower PNI and infection. In addition, glucocorticoid excess is known to cause hematological changes such as leukocytosis with neutrophilia, lymphopenia, monocytopenia, and increased platelet counts (21, 22); these changes may also affect the levels of blood cell-derived inflammatory markers. These mechanisms may help explain why lower PNI and higher inflammatory indices were associated with higher cortisol burden and greater infection risk in our study.

Previous studies in patients with CS have shown that higher cortisol levels are associated with both the development and severity of infection. Consistent with this, Tatsi et al. reported, in a cohort of pediatric patients with CS, that the presence of infection was associated with higher basal cortisol, LNPC, and UFC levels, as well as lower absolute lymphocyte counts (23). Additionally, Sarlis et al. demonstrated an association between severe infections and indices of hypercortisolemia in patients with Ect-CS (24). More severe hypercortisolism has also been associated with higher inflammatory marker levels and lower PNI. In line with this, Detomas et al. reported positive correlations of cortisol after the 1-mg DST with NLR and PLR in patients with endogenous hypercortisolism (25). Likewise, Mangone et al. showed that cortisol after the 1-mg DST correlated positively with NLR and SII and negatively with PNI in both adrenocortical adenoma and adrenocortical carcinoma (26). Similarly, Favero et al. demonstrated that inflammation-based scores, particularly NLR and SII, were associated with cortisol after the 1-mg DST in patients with benign adrenocortical tumors (27). In pediatric endogenous CS, Wurth et al. also showed that NLR and PLR were correlated with basal cortisol, midnight serum cortisol, and UFC (xULN) (28). In this context, our study demonstrated an association between the severity of hypercortisolemia and perioperative infection and further identified significant relationships between cortisol levels, inflammatory indices, and PNI; moreover, to the best of our knowledge, this is the first study to evaluate cortisol-related indices, PNI, and inflammatory indices together in relation to perioperative infection in patients with CS.

DM was another independent predictor of infection in our cohort. This finding is clinically plausible, because DM is common in CS and may further impair immune responses through defects in neutrophil function, cytokine signaling, and humoral immunity (29–31). Severe bone involvement, defined as the presence of fracture(s) or a T-score ≤ -3, also remained independently associated with infection. Rather than reflecting a direct skeletal mechanism contributing to infection, we interpret this variable as a marker of greater disease burden, because severe bone involvement in CS has been associated with more prolonged or more severe cortisol excess (32–34).

CS also adversely affects skeletal muscle health, leading to impaired muscle function (35, 36). In addition, Delivanis et al. reported that higher cortisol levels after the 1-mg DST were associated with lower skeletal muscle area at the L3 level on CT (37). However, evidence regarding the relationship of PNI with sarcopenia in CS remains scarce, although lower PNI has been shown to be independently associated with sarcopenia in population-based adult cohorts (38). In our study, sarcopenia was not evaluated. Further studies are needed to investigate the relationship between PNI and sarcopenia in patients with CS.

The most frequent infections in our cohort were urinary tract infections and pneumonia, which is consistent with previous reports describing urinary tract and respiratory infections among the common infectious complications of CS (39, 40). From a practical standpoint, assessment of 1-mg DST and PNI may represent a simple and inexpensive approach for perioperative risk stratification in CS. Patients with marked hypercortisolism, low PNI, DM, and severe bone involvement may benefit from closer perioperative surveillance and individualized preventive strategies.

This study has several limitations. First, its retrospective design limits causal inference. Second, not all patients underwent the full set of cortisol-related tests, because diagnosis was established once sufficient biochemical criteria were met, resulting in missing data for some secondary hormonal variables. Third, the study was conducted at a single center with a limited sample size. In particular, the very small Ect-CS subgroup may have reduced the reliability of comparisons among the etiological subgroups. Therefore, the subgroup comparison results should be interpreted with caution. Fourth, surgical variables such as operative duration and surgical approach were not consistently available in a standardized manner and therefore could not be incorporated into the multivariable model, although they may influence postoperative infection risk. Fifth, by excluding superficial and non-systemic localized infections, we focused on clinically significant infections; however, this may have led to underestimation of the overall perioperative infection profile. Despite these limitations, our findings provide clinically relevant evidence that hormonal burden, immune-nutritional status, and selected comorbidities jointly contribute to perioperative infection risk in endogenous CS and warrant validation in larger prospective studies.

## 5 Conclusion

Patients with CS are predisposed to infections due to hypercortisolism-related immunosuppression. Identifying reliable predictive markers and risk factors is clinically important. Lower PNI levels, the presence of diabetes mellitus, severe bone involvement, and higher cortisol levels after 1-mg DST may be associated with an increased risk of infection. PNI may serve as a practical and potentially modifiable marker for perioperative risk stratification and targeted preventive strategies in patients with Cushing syndrome.

## Data Availability

The original contributions presented in the study are included in the article/supplementary material. Further inquiries can be directed to the corresponding author.

## Glossary

1-mg DST: 1-mg Overnight Dexamethasone Suppression Test
AUC: Area Under the Curve
Adr-CS: Adrenal Cushing Syndrome
AMI: Acute Myocardial Infarction
BMD: Bone Mineral Density
BMI: Body Mass Index
CS: Cushing Syndrome
DM: Diabetes Mellitus
DVT: Deep Vein Thrombosis
DXA: Dual-Energy X-ray Absorptiometry
Ect-CS: Ectopic Cushing Syndrome
HT: Hypertension
ICU: Intensive Care Unit
LDDST: Low-Dose Dexamethasone Suppression Test
LNPC: Late-Night Plasma Cortisol
LNSC: Late-Night Salivary Cortisol
NLR: Neutrophil-to-Lymphocyte Ratio
NPV: Negative Predictive Value
OR: Odds Ratio
PE: Pulmonary Embolism
PIV: Pan-Immune-Inflammation Value
Pit-CS: Pituitary Cushing Syndrome
PNI: Prognostic Nutritional Index
PPV: Positive Predictive Value
ROC: Receiver Operating Characteristic
SII: Systemic Immune-Inflammation Index
UFC: Urinary Free Cortisol
ULN: Upper Limit of Normal
xULN: Fold Increase Over the Upper Limit of Normal
